# Supplementary material for: All eyes, no IMU: learning flight attitude from vision alone
Source: Npj Robot. 2026 Mar 17;4(1):21. doi: 10.1038/s44182-026-00081-4 (PMC12995713; doi:10.1038/s44182-026-00081-4)
Supplement: Supplementary file 1 — Supplementary Information [file 44182_2026_81_MOESM1_ESM.pdf]

Supplementary Information

S1 Accompanying video

The video accompanying this publication ('npj_experiments.mp4') demonstrates our method in the control-loop of the drone. The first two tests are performed in the Cyberzoo, the flight arena where also the training data was gathered. The last test was performed in a completely different environment to demonstrate robustness to scene variability. From 0:04 to 0:25, the drone receives zero velocity commands (hover), from 0:26-0:57 it receives alternating pitch/roll commands to fly a square pattern, demonstrating that it can also correctly estimate larger angles during flight, from 0:58-1:30 the same pattern was performed but in a previously unseen environment, demonstrating robustness again scene variability.
